# Differential WNT4 expression among various subtypes of moyamoya disease results in alterations of microtubule stability

**DOI:** 10.1002/ctm2.1797

**Published:** 2024-08-04

**Authors:** Shihao He, Zhenyu Zhou, Junze Zhang, Yanru Wang, Ziqi Liu, Xiaokuan Hao, Xilong Wang, Xun Ye, Yuanli Zhao, Rong Wang

**Affiliations:** ^1^ Department of Neurosurgery Beijing Tiantan Hospital, Capital Medical University Beijing China; ^2^ Department of Neurosurgery Peking Union Medical College Hospital, Peking Union Medical College and Chinese Academy of Medical Sciences Beijing China; ^3^ Center of Stroke Beijing Institute for Brain Disorders Beijing China; ^4^ Beijing Institute of Brain Disorders Collaborative Innovation Center for Brain Disorders, Capital Medical University Beijing China

Dear Editor,

Our study characterized the differential protein metabolism among different subtypes of moyamoya disease (MMD). Moreover, overexpression of WNT4 (wingless‐type MMTV integration site family member 4) caused multiple cellular impairments by reducing microtubule stability, which may bring new insight into haemorrhagic MMD.

Moyamoya disease is characterized by occlusion of the intracranial internal carotid artery and the formation of collateral vessels. Intracranial haemorrhage is one of the main initial symptoms and causes of disability in patients with MMD.^1^ Given the obstacles brought by the unknown mechanism of haemorrhagic MMD for diagnosis and treatment, we were motivated to explore the differences in protein between different subtypes of MMD. A study of 170 MMD patients showed that RNF213 A4399T (rs148731719) was closely associated with haemorrhagic MMD.^2^ However, this study only explored the differences of RNF213 between MMD subtypes at the gene level without validation in vitro, so there is no evidence of any physiological impairment. In this study. We not only used proteomic profiling to identify the differences in protein metabolism among different subtypes of MMD but also explored the potential mechanisms of haemorrhagic MMD by in vitro experiments.

In the discovery group, the DIA quantification proteomics was then used to identify protein metabolic changes and potential biomarkers in different subtypes of MMD (Table S1). Proteins with significantly different expression levels were listed and functional enrichment analysis was performed (Figure 1B, Table S2). Notably, WNT4 was not only one of the proteins with the most significant differential levels but also was found to be closely related to various physiological functions (Figure 1C).

**FIGURE 1:**
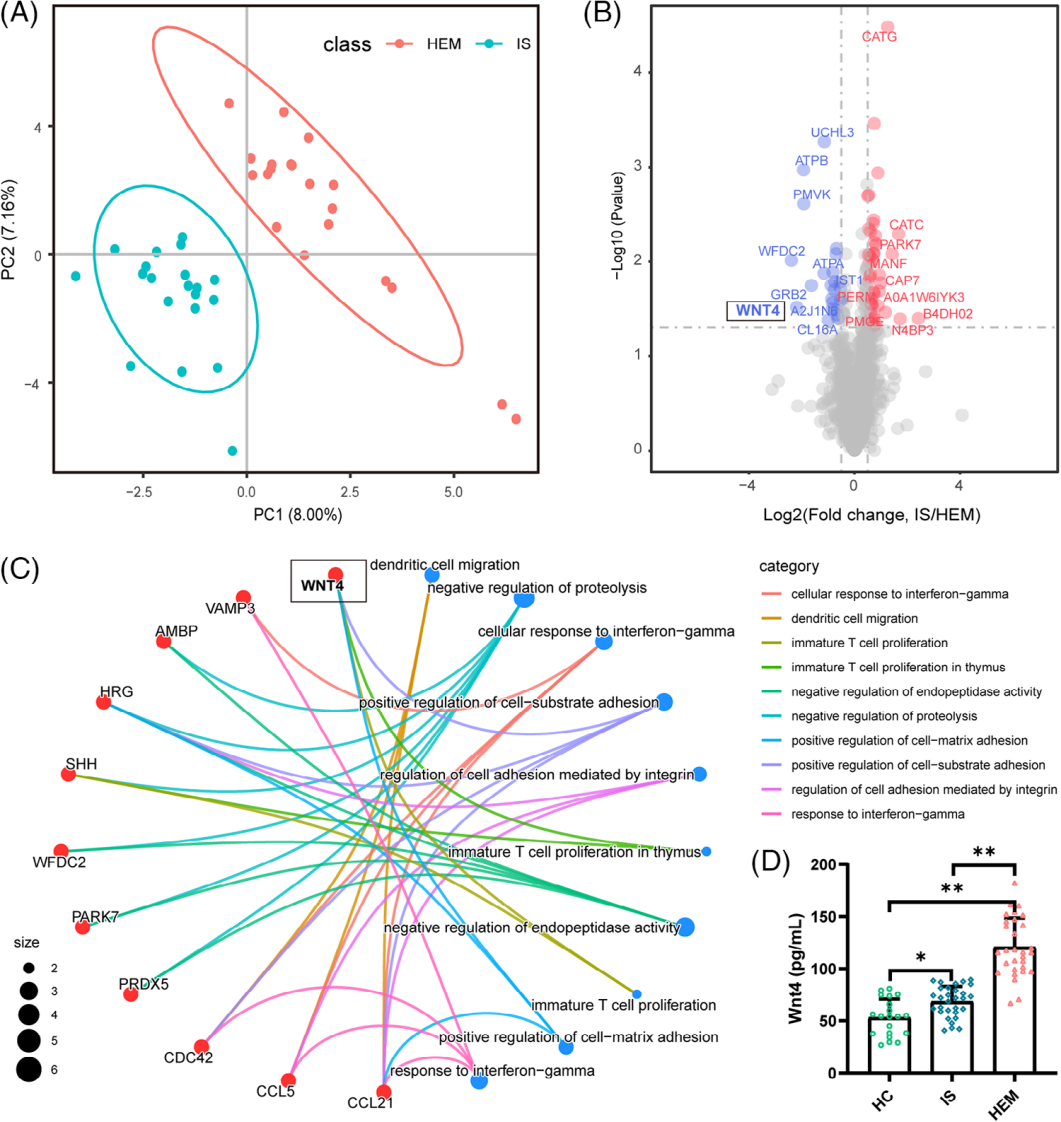
Differential analysis and validation of genes for haemorrhagic and ischemic MMD. (A) Principal component analysis (PCA) of the gene data in HEM (*N* = 20) and IS(*N* = 20) groups. (Red: HEM group; Blue: IS group). (B) Volcano plot of differentially expressed genes. (Red: upregulation; blue: downregulation). (C) Chordal graph showing the interconnection of the differentially expressed genes. (D) The expression of WNT4 in HC, IS, and HEM serum from different patients. The level of WNT4 was measured by ELISA assay. The abscissa represents the grouping. The ordinate indicates the expression level of WNT4. Results were mean ± SD for HC (*N* = 20), IS (*N* = 30), and HEM (*N* = 30). *p < 0.05, **p < 0.01.

WNT4, a member of the WNT family located in chromosome 1p36, normally exerts cellular physiological effects through WNT/β‐catenin signalling that was observed to be abnormally elevated in thoracic aortic aneurysms, a typical haemorrhagic vascular disease.^3^ This suggests that overexpression of WNT4 in haemorrhagic MMD may be a new insight into the mechanism. In the validation group, the results of serum ENLISA in the validation group showed that the WNT4 level in HEM (haemorrhagic moyamoya disease) was significantly higher than that in the HC (health controls) and IS (ischemic moyamoya disease), which was consistent with our previous results (Figure 1D, Table S1). We then explored the possible physiological impairment caused by overexpression of WNT4 in haemorrhagic MMD using a variety of in vitro experiments. In immunofluorescence staining, compared with the oe‐Vector group, the density of microtubules and cytoskeleton in the oe‐WNT4 group was significantly decreased (Figure 2A). Compared with the oe‐Vector group, the stability of microtubules in the oe‐WNT4 group was significantly reduced by measuring the degree of acetylation (Figure 2C). Considering that this may be the key link between WNT4 and haemorrhagic MMD, we further investigated the WNT4‐related signalling pathway to elucidate the relationship between WNT4 overexpression and microtubule abnormalities. The result of the Western blot showed that although the total tau (Tubulin binding protein) expression level was unchanged, p‐tau(phosphorylated tau)was increased in the oe‐WNT4 group (Figure 2B). This suggests that overexpression of wnt4 may cause an increase in tau phosphorylation. Tau is a microtubule‐binding protein that binds to microtubules through its specific domain.^4^ Previous studies have shown that Wnt/ beta‐catenin signaling interacts with presenilin 1 and that levels of tau phosphorylation are significantly higher in cerebrospinal fluid in patients with Alzheimer's disease.{Ref. 5 and 6} Although tau is not a structural component of microtubules, hyperphosphorylation can disrupt tua function, leading to microtubule instability and even breakdown.

**FIGURE 2:**
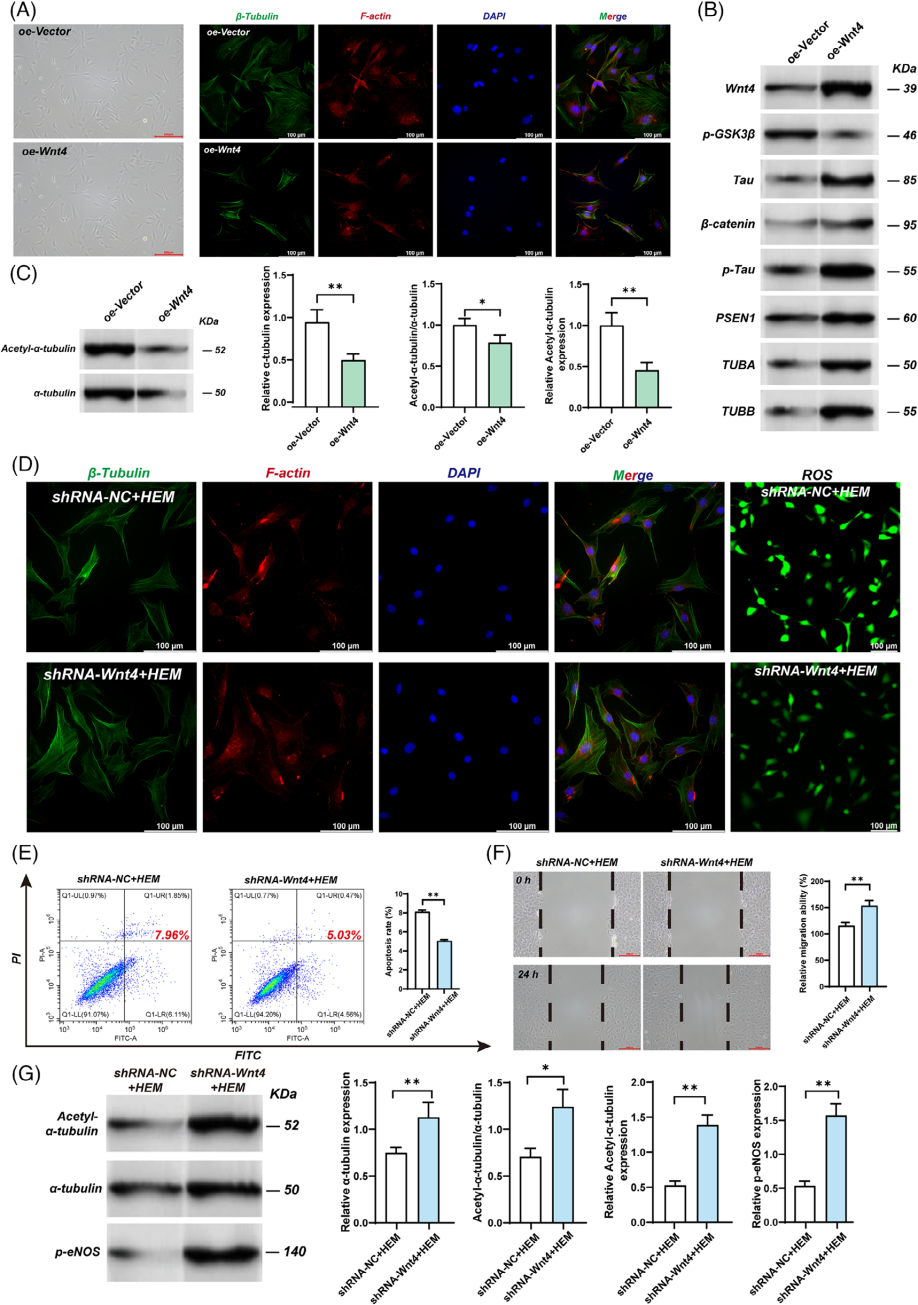
The effects of the expression and knockout of WNT4 on HBVSMC cells. HBVSMC cells were treated with oe‐Vector or oe‐WNT4. Furthermore, HBVSMC cells were treated with shRNA‐NC or shRNA‐WNT4, then cultured in SMCM medium with 2% FBS for 24 h, and then cultured in DMEM medium with 2.5% HEM serum for 24 h. (oe‐Vector: Control group. oe‐WNT4: WNT4 overexpression group. shRNA‐NC+HEM: HBCSMC cultured with HEM group serum without knockdown of WNT4. shRNA‐WNT4+HEM: HBCSMC cultured with HEM group serum with knockdown of WNT4.). (A) The cell morphology in each group was taken under a light microscope. Bar = 200 µm. HBVSMC cells were treated with oe‐Vector or oe‐WNT4. The microtubule density in each group was detected by immunofluorescence. Bar = 100 µm. (Green: tubulin; red: actin; blue: cell nucleus; mix: a stack of the three). (B) HBVSMC cells were treated with oe‐Vector or oe‐WNT4. oe‐Vector: Control group. oe‐WNT4: WNT4 overexpression group. The expression of WNT4, p‐GSK3β, Tau, β‐catenin, p‐Tau, PSEN1, TUBA, and TUBB from the indicated group was detected by western blot assay. (C) The expression of Acetyl‐α‐tubulin and α‐tubulin from the indicated group was detected by western blot assay. oe‐Vector: Control group. oe‐WNT4: WNT4 overexpression group. Results were mean ± SD for three individual experiments. The microtubule stability was measured by the acetylation degree of α‐tubulin. Results were mean ± SD for three individual experiments. *p < 0.05, **p < 0.01. (D) The microtubule density in each group was detected by immunofluorescence. The ROS levels were detected by DCFH‐DA probe fluorescence microscopy. Bar = 100 µm. (E) Flow Cytometric Analysis of cell apoptosis in HBVSMC cells. Results were mean ± SD for three individual experiments. *p < 0.05, **p < 0.01. (F) Cell migration was detected by scratch assay. Bar = 200 µm. Results were mean ± SD for three individual experiments. *p < 0.05, **p < 0.01. (G) The expression of Acetyl‐α‐tubulin and α‐tubulin from the indicated group was detected by western blot assay. The microtubule stability was measured by the acetylation degree of α‐tubulin. Results were mean ± SD for three individual experiments. *p < 0.05, **p < 0.01.

Microtubules are important components of the cytoskeleton, which function as tracks for intracellular transport.^7^ Given the critical role of microtubule stability for vascular integrity, we provided in vitro evidence of the cellular physiological impairment caused by microtubule abnormalities in haemorrhagic MMD. The Western blot results showed that the proportion of activated eNOS(endothelial nitric oxide synthase)of the oe‐WNT4 group was significantly reduced (Figure S1B). The reduction of activated eNOS will inevitably lead to the reduction of NO, an important cytokine to maintain the normal function of VSNC, which will cause vascular injury.^8^ In addition, flow cytometry and cell migration assay showed that overexpression of WNT4 also increased cell apoptosis and decreased cell migration (Figure S1C,D). It has previously been shown that a specific increase in ROS in haemorrhagic MMD indicates the abnormal function of cellular mitochondria.^9^ In contrast, consistent results were observed in our study (Figure S1A). This suggests that microtubule abnormalities caused by overexpression of WNT4 may be involved in the pathogenesis of haemorrhagic MMD through multiple cellular impairment mechanisms.

Immunofluorescence results showed that the density of microtubules and cytoskeleton was restored after WNT4 knockdown by shRNA (Figure 2D). At the same time, the degree of acetylation of microtubules was also significantly increased compared with the shRNA‐NC+HEM, suggesting that the stability of microtubules was restored to a certain extent after WNT4 knockdown (Figure 2G). The levels of activated eNOS, apoptosis, cell migration, and ROS were nearly normalized after the WNT4 knockdown (Figure 2D–G). In addition, DDK‐1(Dickkopf‐1), a selective inhibitor of WNT4, improved microtubule density and alleviated other cell dysfunctions in the oe‐WNT4 group (Figure 3; Figure S2). This further confirmed that overexpression of WNT4 could cause microtubule abnormalities and other cellular dysfunctions in haemorrhagic MMD.

**FIGURE 3:**
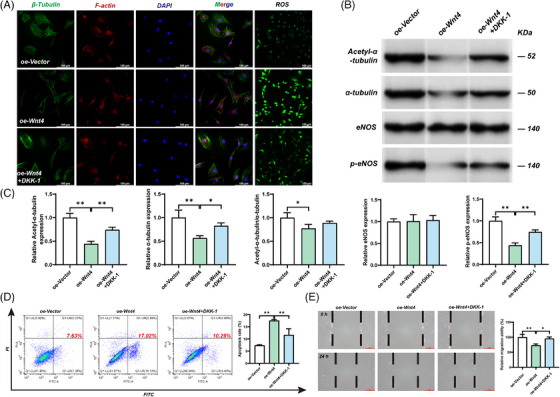
The effect of WNT4 overexpression on HBVSMC cells after the addition of inhibitor DKK‐1. HBVSMC cells were treated with oe‐Vector or oe‐WNT4, and then one group of cells transfected with oe‐WNT4 was cultured in SMCM medium with 100 µg/L DDK‐1. (oe‐Vector: Control group. oe‐WNT4: WNT4 overexpression group. oe‐WNT4+DDk‐1: WNT4 overexpression group with inhibitor DDK‐1). (A) The microtubule density in each group was detected by immunofluorescence. The ROS levels were detected by DCFH‐DA probe fluorescence microscopy. Bar = 100 µm. (B, C) The microtubule stability was measured by the methylation degree of α‐tubulin. The expression of Acetyl‐α‐tubulin, α‐tubulin, eNOS, and p‐eNOS was detected by western blot assay. Results were mean ± SD for three individual experiments. *p < 0.05, **p < 0.01. (D) Flow cytometric analysis of cell apoptosis in HBVSMC cells Results were mean ± SD for three individual experiments. *p < 0.05, **p < 0.01. (E) Cell migration was detected by scratch assay. Bar = 200 µm. Results were mean ± SD for three individual experiments. *p < 0.05, **p < 0.01.

To date, the biomarkers and mechanisms of haemorrhagic moyamoya disease are unknown. Our study suggests that overexpression of WNT4 may lead to decreased stability of cellular microtubules, which promotes rupture and haemorrhage of the moyamoya vessel (Figure 4). This study provides a theoretical foundation for the strategy of biomarkers for moyamoya disease typing, which sheds light on mechanistic studies of haemorrhagic MMD.

**FIGURE 4:**
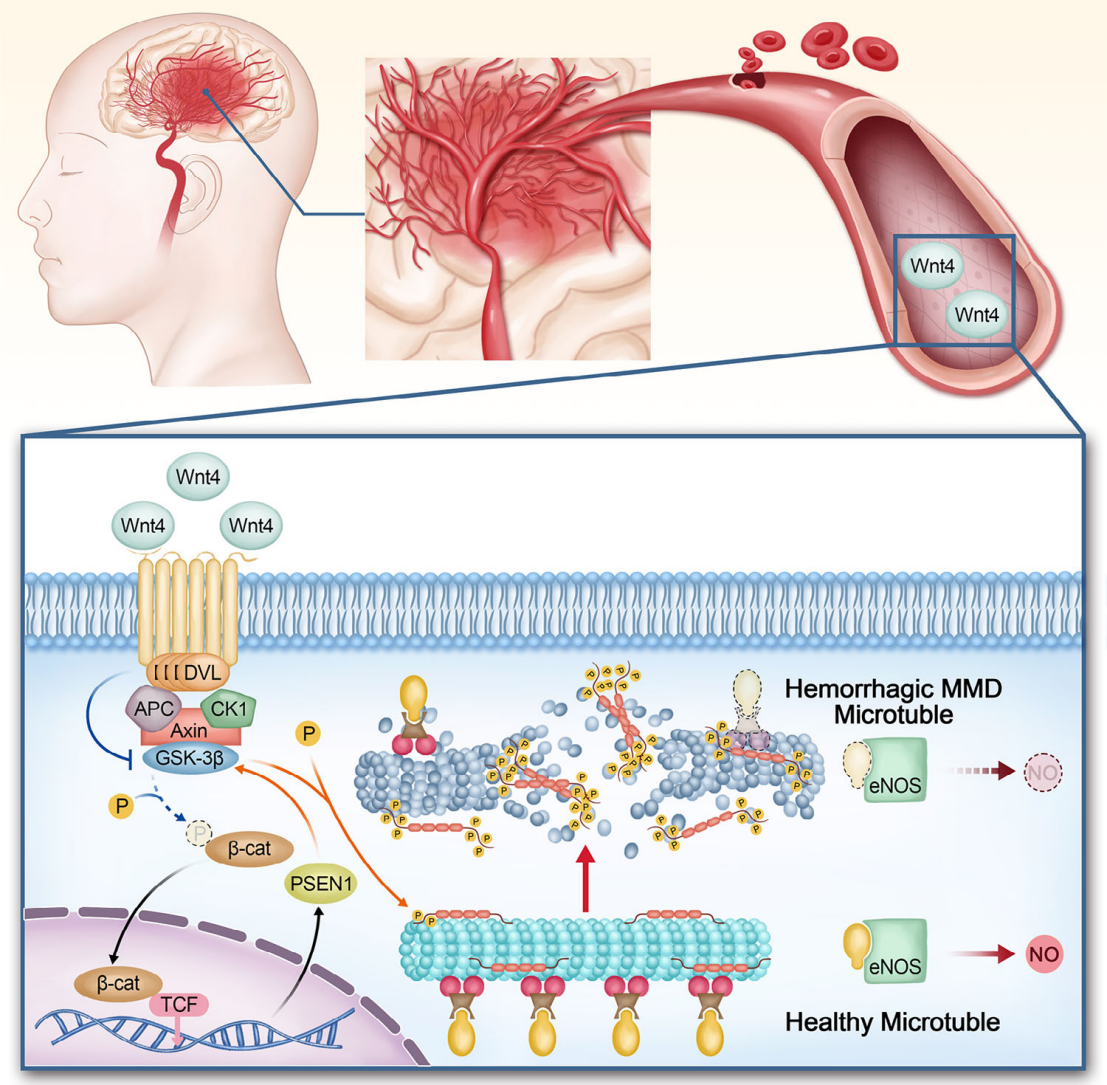
Mechanism of microtubule dysfunction caused by overexpression of WNT4 in haemorrhagic moyamoya disease. Overexpression of WNT4 upregulates the WNT/β‐catenin signalling pathway, resulting in increased phosphorylation of TAU. Hyperphosphorylated TAU loses its ability to stabilize microtubules, which leads to a decline in the stability of cell microtubules and their subsequent disintegration. This results in multiple cellular physiological damage, such as a decrease in activated eNOS, which induces a decrease in NO. β‐cat, β‐catenin; APC, adenomatous polyposis coli gene; CK1, casein kinase 1; DLV, dishevelled segment polarity protein; eNOS, endothelial nitric oxide synthase; GSK‐3β, glycogen synthase kinase 3β; MMD, moyamoya disease; NO, nitric oxide; P, phosphate group; PSEN1, presenilin 1; TCF, T‐cell factor; WNT4, wingless‐type MMTV integration site family member 4.

## AUTHOR CONTRIBUTIONS

Shihao He, Yuanli Zhao, and Rong Wang conceived and designed the experiments. Shihao He, Zhenyu Zhou, Junze Zhang, Yanru Wang, Ziqi Liu, Xiaokuan Hao, and Xilong Wang performed experiments. Yuanli Zhao, Rong Wang, and Xun Ye contributed reagents, materials, and analytical tools. Shihao He, Zhenyu Zhou, Junze Zhang, Yanru Wang, Yuanli Zhao, and Rong Wang wrote the manuscript.

## CONFLICT OF INTEREST STATEMENT

The authors declare no conflict of interest.

## ETHICS STATEMENT

This study was approved by the Institutional Ethics Committee of the Beijing Tiantan Hospital, Beijing, China (KY 2020‐045‐02).

## Supporting information

Supporting information

## Data Availability

The data presented in the current study are available from the corresponding author upon reasonable request.

## References

[1] Scott RM , Smith ER . Moyamoya disease and moyamoya syndrome. N Engl J Med. 2009;360(12):1226‐1237. doi:10.1056/NEJMra0804622 19297575

[2] Toft M , et al. Molecular analysis of RNF213 gene for moyamoya disease in the Chinese Han population. PLoS One. 2012;7. doi:10.1371/journal.pone.0048179 PMC3479116 23110205

[3] Kostina A , Bjork , H , Ignatieva E , et al. Notch, BMP and WNT/β‐catenin network is impaired in endothelial cells of the patients with thoracic aortic aneurysm. Atheroscler Suppl. 2018;35:e6‐e13. doi:10.1016/j.atherosclerosissup.2018.08.002 30172576

[4] Kosik KS . The molecular and cellular biology of tau. Brain Pathol. 1993. doi:10.1111/j.1750-3639.1993.tb00724.x 8269082

[5] Kengo Uemura NK . 1 Ryuichi Kohno,1 Akira Kuzuya,1 & Takashi Kageyama, H. S., 1 and Shun Shimohama1*. Presenilin 1 mediates retinoic acid‐induced differentiation of SH‐SY5Y cells through facilitation of WNT signaling. J Neurosci Res. 2003;73(2):166‐175. doi:10.1002/jnr.10641 12836159

[6] Miller BL , et al. APP, PSEN1, and PSEN2 mutations in early‐onset Alzheimer disease: a genetic screening study of familial and sporadic cases. PLoS Med. 2017;14:e1002270. doi:10.1371/journal.pmed.1002270 28350801 PMC5370101

[7] Goodson HV , Jonasson EM . Microtubules and microtubule‐associated proteins. Cold Spring Harb Perspect Biol. 2018;10:a022608. doi:10.1101/cshperspect.a022608 29858272 PMC5983186

[8] Hussong SA , Banh AQ , Van Skike CE , et al. Soluble pathogenic tau enters brain vascular endothelial cells and drives cellular senescence and brain microvascular dysfunction in a mouse model of tauopathy. Nat Commun. 2023;14:2367. doi:10.1038/s41467-023-37840-y 37185259 PMC10126555

[9] Wang X , Han C , Jia Y , Wang J , Ge W , Duan L . Proteomic profiling of exosomes from hemorrhagic moyamoya disease and dysfunction of mitochondria in endothelial cells. Stroke. 2021;52:3351‐3361. doi:10.1161/strokeaha.120.032297 34334053

