# Supplementary material for: Differential WNT4 expression among various subtypes of moyamoya disease results in alterations of microtubule stability
Source: Clin Transl Med. 2024 Aug 4;14(8):e1797. doi: 10.1002/ctm2.1797 (PMC11298546; doi:10.1002/ctm2.1797)
Supplement: Supplementary file 1 — Supporting information [file CTM2-14-e1797-s001.pdf]

Supplementary materials

Differential WNT4 expression among various subtypes of moyamoya disease results in alterations of microtubule stability

Shihao He#,[1, 2] MD, PhD; Zhenyu Zhou,[#1] MD; Junze Zhang,[1] MD, PhD; Yanru Wang,[1] MD; Ziqi Liu,[1] MD, PhD; Xiaokuan Hao,[1] MD; Xilong Wang,[1] MD; Xun Ye,[1] MD, PhD; Yuanli Zhao,*[1, 2, 3, 4] MD, PhD; Rong Wang,*[1, 3, 4] MD, PhD

[1]Department of Neurosurgery, Beijing Tiantan Hospital, Capital Medical University, Beijing 100070, China

[2] Department of Neurosurgery, Peking Union Medical College Hospital, Peking Union Medical College and Chinese Academy of Medical Sciences; Beijing, 100730, China.

[3]Center of Stroke, Beijing Institute for Brain Disorders, Beijing 100069, China

[4]Beijing Institute of Brain Disorders, Collaborative Innovation Center for Brain Disorders, Capital Medical University, Beijing, 100069, China

#These authors contributed equally to this work.

***Corresponding authors:**

Yuanli Zhao; E-mail: zhaoyuanli@126.com, Telephone: +86 13801121203

Rong Wang; E-mail: ronger090614@126.com, Telephone: +86 13001190333

Methods

Data available statement

The DIA (data-independent acquisition mass spectrometry) quantification proteomics data has been uploaded to the ProteomeXchange Consortium via the iProX partner repository (subject ID is IPX0005455000) with the dataset identifier PXD038468. Additional data will be made available upon reasonable request to the corresponding authors.

Participant enrollment

Digital subtraction angiography of cerebral vascular was performed in all patients. MMD diagnosis were performed according to the guidelines[1]. Peripheral blood samples were taken from all patients and healthy controls. Written informed consent was obtained from all participants (parents/guardians of patients aged <18 years). This study was approved by the Institutional Ethics Committee of the Beijing Tiantan Hospital, Beijing, China (KY 2020-045-02).

Participant enrollment

Digital subtraction angiography of cerebral vascular was performed in all patients. MMD diagnosis were performed according to the guidelines. Peripheral blood samples were taken from all patients and healthy controls. Written informed consent was obtained from all participants (parents/guardians of patients aged <18 years). This study was approved by the Institutional Ethics Committee of the Beijing Tiantan Hospital, Beijing, China (KY 2020-045-02).

Protein profile analysis

DIA quantification proteomics was used to identify differences in protein expression between patient sera from the HEM (hemorrhagic moyamoya disease), IS (ischemic moyamoya disease) and HC (health controls) groups. The protein samples were denatured by adding protein lysate to serum samples at a ratio of 1:9 for samples and protein lysate (w/w), and dithiothreitol (DTT) (1M final concentration) was added to the serum samples for 30 min at 37°C. After the samples were cooled to room temperature, 0.55 M iodoacetamide was added to the samples, which were incubated for 30 min at room temperature in the dark. The proteins were then enriched using an SPE C18 column. After extraction and purification, protein samples were redissolved

in 25 μL of 50 mM NH_4HCO_3, vortexed for 1 min, and then centrifuged in a microcentrifuge for 1 min. Protein concentration in the supernatants was determined using Bradford reagent. The purity was confirmed by sodium dodecyl-sulfate polyacrylamide gel electrophoresis (SDS-PAGE). The protein was then digested by adding trypsin to the protein solution at a ratio of 1:20 for enzymes and proteins (w/w) and incubating for 14–16 h at 37°C. For high-pH RP separation, an equal amount of peptide from each sample was pooled into a mixture, and 20 μg of the mixture was diluted with 2 ml of mobile phase A (5% ACN, pH 9.8) for injection. High-pH RP separation was performed using a Shimadzu LC-20AB HPLC system (Shimadzu, Kyoto, Japan) coupled with a Gemini high-pH C18 column. Finally, all the fractions were combined into a total of 10 fractions, which were then frozen and dried. The peptides separated by liquid phase chromatography were ionized by a nanoESI source (Advion, Triversa Nanomate) and then passed to a tandem mass spectrometer Oritrap Exploris 480 (Thermo Fisher Scientific, San Jose, CA) for data-dependent acquisition (DDA) library construction detection and DIA mass spectrometry detection. For bioinformatics analysis, DDA data were identified using the Andromeda search engine within MaxQuant for spectral library construction. For large-scale DIA data, the mProphet algorithm was used to complete analytical quality control and to provide a large number of reliable quantitative results. Gene ontology (GO), Kyoto Encyclopedia of Genes and Genomes (KEGG), and pathway functional annotation analyses were also performed. Based on the quantitative results, differences in protein expression between various groups were found, and finally, function enrichment analysis and protein-protein interaction maps of the differentially expressed proteins were performed. The protein interaction relationship was analyzed using the STRING software (version 3.2.1, https://string-db.org/). Bioinformatics analysis was performed using the R software (version 3.4, R Foundation for Statistical Computing). The DIA analysis was performed by Beijing Genomics Institute (BGI, Shenzhen, China).

Blood samples

Peripheral blood samples were collected from all patients by venipuncture using a

CPT Vacutainer containing sodium citrate as an anticoagulant (BD Biosciences, Heidelberg, Germany) prior to revascularization surgery. Blood samples were collected from healthy adult controls as described above. The Vacutainers were centrifuged for 8 min at 460 g. The serum with lymphocytes was transferred into a new Falcon tube (352096, Corning) and centrifuged again at 3000 r/min for 15 min. The serum supernatant was removed and stored in cryovials at –80°C prior to use.

Enzyme-linked immunosorbent assay

Enzyme-linked immunosorbent assay was used to detect Wnt4 expression levels in serum using Human Wnt4 ELISA Kit (P56705) (RayBiotech, Guangzhou, China). Configure the standards according to the reagent instructions, the TNF-α standard concentrations are in order: 1500, 750, 375, 187.5, 94, 47, 23.5, 0 pg/mL. refer to the analytical layout table to determine the number of wells to be used and place the remaining wells and desiccant back into the bag and seal the ziplock bag. Store the unused wells at 4°C. Add 100 μL of standard and sample to each well. Cover with the supplied adhesive strip. incubate at 37°C for 2 hours. Remove the liquid from each well and do not wash. Add 100 μL (1x) of biotin antibody per well. Cover with new adhesive strips. Incubate at 37°C for 1 hr. Aspirate each well and wash, repeat the process twice for a total of three washes. Add 100 μL (1x) of enzyme labeled affinity protein to each well. Cover the microtitre plate with a new adhesive strip. Incubate at 37°C for 1 hr. Repeat the aspiration/washing process 5 times as in step 6. Add 90 μL of TMB substrate per well. Incubate at 37°C for 15-30 minutes, protected from light. Add 50 μL of termination solution per well to terminate the reaction. Immediately measure the optical density (OD) of each well at 450 nm using an enzyme meter.

Cell culture and treatment

The human cerebrovascular smooth muscle cell line HBVSMCs was purchased from ScienCell (ScienCell Research Laboratories, Hubei, China). HBVSMCs cells were cultured in passaging under the conditions of Smooth Muscle Cell Growth Supplement (SMCGS, 1152, SCIENCELL RESEARCH LABORATORIES), which contains 1% P/S double antibody, 1% smooth muscle cell growth supplement (SMCGS, 1152, SCIENCELL RESEARCH LABORATORIES) and 2% fetal bovine

serum (FBS, SH30070.03, Hyclone) in smooth muscle cell medium (SMCM, 1152, ScienCell Research Laboratories). When cells were fused to 90%, the old medium was discarded. The cells were washed twice with 2 mL of PBS, after discarding PBS, 2 mL of 0.25% trypsin (15090046, Gibco, Grand Island)-0.02% EDTA mixed digest was added. The cells were placed under the microscope for about 30 s. When the cells became rounded, the digestion was terminated by quickly adding 2 mL of complete culture medium When the cells became round, add 2 mL of complete medium quickly to terminate the digestion, blow gently and collect the cells. 800 rpm, 4℃, centrifuge for 5 min, discard the supernatant, resuspend the cells with complete medium, culture in separate flasks, and change the solution every other day.

Lentiviral vector construction

For the full-length sequence of CDS region of Wnt4, specific primers were designed, and according to the polyclonal site of pLVX-Puro vector, Spe I (ACTAGT) (1086A, Takara)and Xba I (TCTAGA) (1093A, Takara)were added upstream and downstream of the primers respectively, and the PCR products with Spe I and Xba I were purified and recovered. The sequences of the primers for Wnt4 were Forward: 5'-GACTAGTATGAGTCCCGCTCGTG -3'; Reverse: 5'-GCTCTAGATCATCGGCACGTGTGTGCAAC -3'. The pLVX-Puro lentiviral overexpression plasmid is shown below:

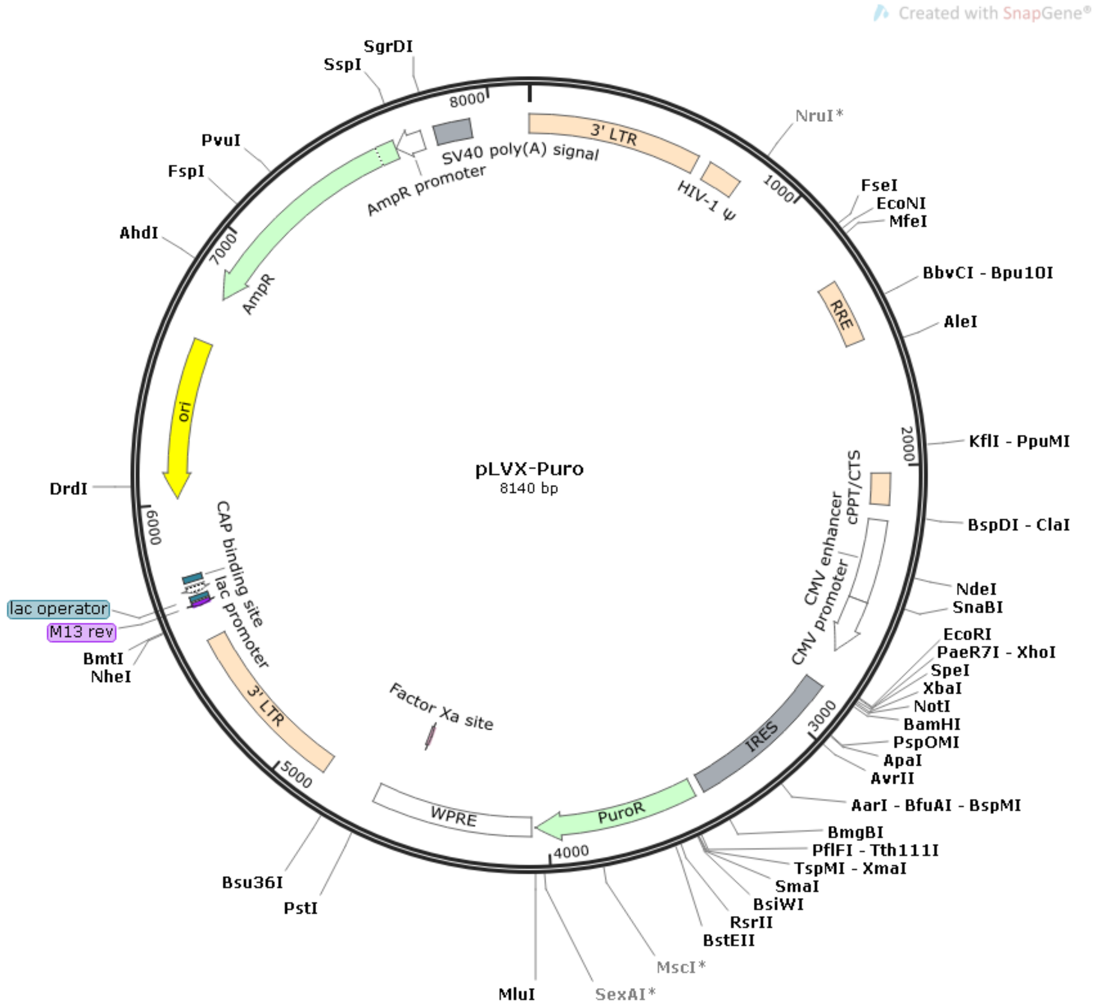

Prepare 50 μL of digestion system according to the table below. Add the reagents in the order of the table, mix with a pipette, centrifuge briefly, and react at 37°C for 3 h. Perform agarose gel electrophoresis on the vector digest products to recover the target bands.

Reagents	Volumetric (μL)
ddH_2O	41
10×CutSmart Buffer2	5
Purified plasmid DNA (1 μg/μL)	2
Spe I (10 U/μL)	1

Xba I (10 U/μL)	1
Total	50

The double-enzymatic linearized vector and annealed double-stranded DNA were ligated by T4 DNA ligase (2011A， Takara) at 16°C overnight. The reaction system was as follows:

Reagents	Volumetric(μL)
Linearized carriers (100 ng/μL)	1
Double-stranded DNA (100 ng/μL)	1
10×T4 DNA ligase Buffer	2
T4 DNA ligase	1
ddH_2O	Complement to20

Add 10 μL of reaction product to 100 μL of sensory cells, flick the wall of the tube to mix well. The cells were placed on ice for 30 min, then heat-excited at 42°C for 90 s, and finally incubated in an ice-water bath for 2 min. 500 μL of LB medium was added, and the cells were incubated in a shaking bed at 37°C for 1 h. Appropriate amount of bacterial solution was evenly spread on a plate containing Amp antibiotics, and the cells were inverted and incubated in an incubator for 16 h. The cells were then incubated for 1 h at 37°C.

According to the principle of RNAi sequence design, the target sequence was designed for the sequence of the target gene Wnt4, and the DNA sequence containing the target site and the cleavage site was synthesized. The synthesized primers were dissolved in annealing buffer, and then cooled naturally to room temperature after 15 minutes in a 90°Cwater bath.

shRNA-Wnt4#1plasmid

(sequence:5'CCGGCACGCACTGAAGGAGAAGTTTCTCGAGAAACTTCTCCTT

CAGTGCGTGTTTTTG -3');

shRNA-Wnt4#2plasmid

(sequence:5'CCGGGCACTGAAGGAGAAGTTTGATCTCGAGATCAAACTTCTCCTTCAGTGCTTTTTG -3');

shRNA-Wnt4#3 plasmid

(sequence:5'CCGGGTCAGGATGCTCTGACAACATCTCGAGATGTTGTCAGAGCATCCTGACTTTTTG -3');

shRNA-NC control plasmid

(sequence:5'CCGGCAACAAGATGAAGAGCACCAACTCGAGTTGGTGCTCTTCATCTTGTTGTTTTTG -3').

The carriers are shown below:

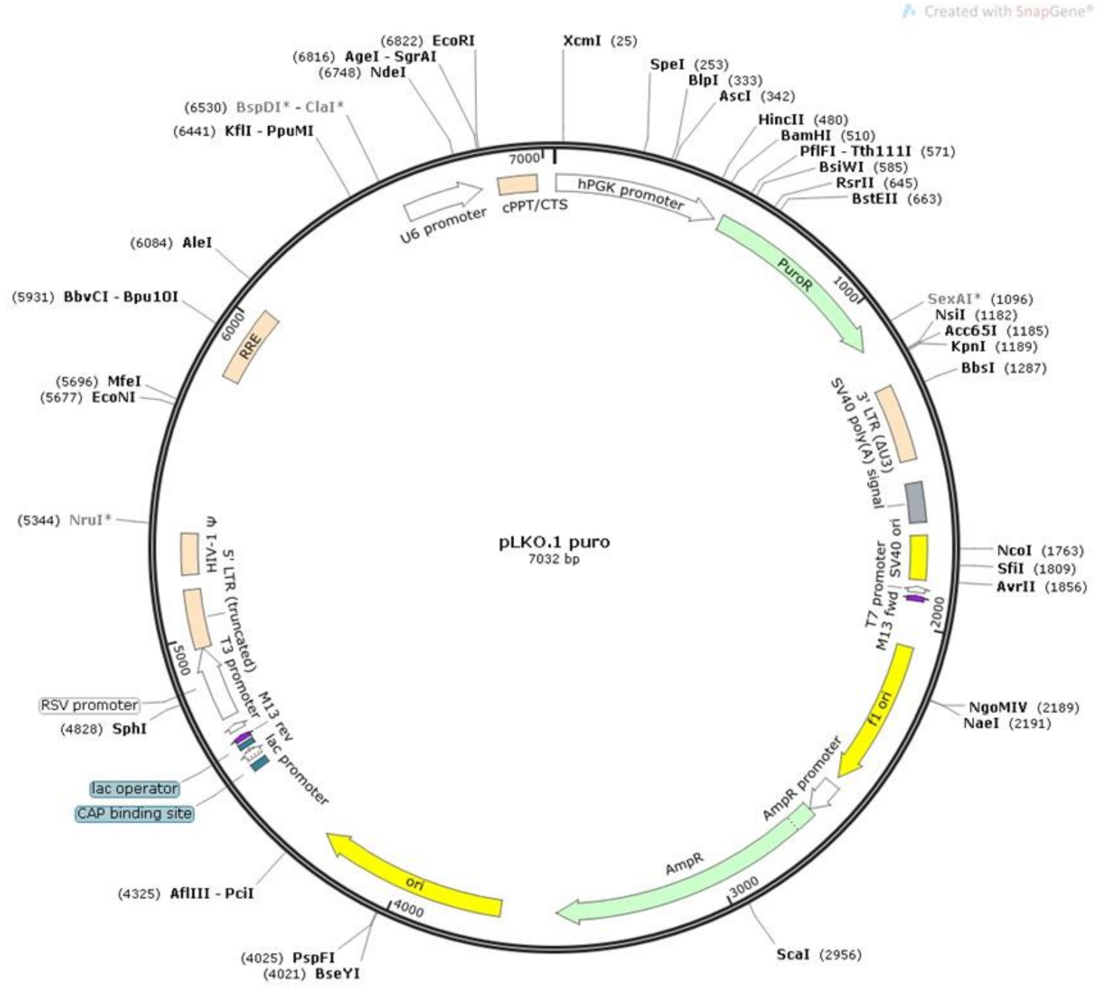

Prepare 50 μL of digestion system according to the table below. The reagents were added sequentially in the order listed and mixed with a pipette. After brief centrifugation, the reaction was carried out at 37°C for 3 h. Agarose gel electrophoresis was performed on the vector digest products to recover the target bands.

Reagents	Volumetric(μL)
ddH_2O	41
10×CutSmart Buffer2	5
Purified plasmidsDNA (1 μg/μL)	2
Age I (10 U/μL)	1
EcoR I (10 U/μL)	1
Total	50

The double-enzymatically linearized vector and annealed double-stranded DNA were ligated by T4 DNA ligase overnight at 16°C. The reaction system was as follows:

Reagents	Volumetric(μL)
Linearized carriers (100 ng/μL)	1
Double-stranded DNA (100 ng/μL)	1
10×T4 DNA ligase Buffer	2
T4 DNA ligase	1
ddH_2O	Complement to20

Add 10 μL of reaction product to 100 μL of sensory cells and mix well. The cells were placed on ice for 30 min, heat-excited at 42°C for 90 sec, and incubated in an ice-water bath for 2 min. Add 500 μL of LB medium and incubate for 1 h at 37°C with shaking bed. Spread the appropriate amount of bacterial solution onto the plate containing Amp antibiotic, and incubate for 16 h in an inverted position in a constant temperature incubator.

The following reaction system was prepared, shaken and mixed, and centrifuged briefly. In an ultra-clean bench, single colonies were picked into 20 μL of the identification system with a sterile tip, mixed well, and placed in a PCR instrument for reaction.

Reagents	Volumetric(μL)
ddH_2O	9.2
2×Taq Plus Master Mix	10
Upstream primers (10 μM)	0.4
Downstream primers (10 μM)	0.4
Single colony	-
Total	20

PCR reaction conditions:

Step1	94°C	3 min
Step2 22 cycles	94°C	30 s
	55°C	30 s
	72°C	30 s
Step3	72°C	5 min
Step4	4°C	

The identified positive clones were inoculated in appropriate amount of LB liquid medium containing Amp antibiotic and incubated at 37°C for 16 h. Appropriate amount of bacterial fluid was taken for sequencing.

The correctly sequenced bacterial fluids were inoculated in 10 mL of LB liquid medium containing Amp antibiotics and incubated overnight at 37°C. The bacterial fluids were collected from the cells by centrifugation at 12000 rpm for 2 min. Collect the overnight culture in a labeled 5 mL centrifuge tube and centrifuge at 12000 rpm for 2 min to collect the bacteria; discard the supernatant, add 250 μL of cell resuspension solution,

and shake well to make the bacterial clusters suspend evenly; add 250 μL of cell lysate, and then add 10 μL of Proteinase K. Upside down for 5-6 times, and mix well; let it stand for 1-2 min, so as to make the bacterial body clear and lysed; add 350 μL of neutralization solution, and mix well, so as to make the protein completely precipitate out. Add 350 μL of neutralizing solution. Add 350 μL of Neutralization Solution. Mix up and down until the protein is completely precipitated, and leave on ice for 5 minutes; centrifuge at 10,000 rpm for 10 minutes and discard the protein. Collect the supernatant in another clean, sterile 1.5 mL EP tube; centrifuge at 2000 rpm for 5 minutes while preparing a labeled recovery column. Transfer the supernatant to the recovery column and centrifuge at 12,000 rpm for 1 minute; discard the lower reject; add 600 μL of pre-configured rinse solution and centrifuge at 12,000 rpm for 1 minute. Dispose of the lower waste solution, repeat the process and centrifuge at 12000 rpm for 2 min to further remove the residual rinse solution; transfer the recovery column to a new 1.5 mL EP tube in an ultra-clean bench, let it stand for 10 min, and let it dry naturally; add 95 μL of Nuclease-Free Water to the recovery column, let it stand for 2 min, and centrifuge at 12000 rpm for 2 min. The samples were collected and numbered as oe-Vector, oe-Wnt4, shRNA-Wnt4#1, shRNA-Wnt4#2, shRNA-Wnt4#3, shRNA-NC.

24 h before transfection, 239 T cells in logarithmic growth phase were digested with trypsin. The cell density was adjusted to 5×106 cells/15 mL with medium containing 10% serum, and re-inoculated in 10 cm2 cell culture dishes at 37°C with 5% CO2. When the cell density reached 70%~80%, it could be used for transfection.

Change to serum-free medium 2 h before transfection. Prepare a sterilized centrifuge tube, add 20 μg of vector plasmid, 15 μg of pHelper 1.0 vector plasmid and 10 μg of pHelper 2.0 vector plasmid, and mix well with the corresponding volume of Giket transfection reagent. Adjust the total volume to 1 mL and incubate at room temperature for 15 minutes.

The mixture was slowly added dropwise to 293T cell culture medium, mixed well, and incubated at 37°C with 5% CO2. Discard the medium containing the transfection mixture after 6 h of incubation. Wash once by adding 10 mL of PBS solution, and shake the culture dish gently to wash the residual transfection mixture and then discard it.

Slowly add 20 mL of cell culture medium containing 10% serum and incubate for 48 h at 37°C with 5% CO2.
The supernatant of 293T cells 48 h after transfection was collected and centrifuged at 4°C, 4000 g for 10 min to remove cell debris. The supernatant was added to a 0.45 μm filter in a 40 mL ultracentrifuge tube, and centrifuged at 25000 rpm, 4°C for 2 h. At the end of centrifugation, the supernatant was discarded, and the liquid remaining on the wall was removed as much as possible, and the viral preservation solution was added, and gently and repeatedly blown.
After fully dissolved, 10000 rpm, centrifugation for 5 min, take the supernatant.
The day before the assay, 293T adherent cells were used to spread the plate (96 wells). Each well had 4×104 cells in a volume of 100 μL. 10 sterile EP tubes were prepared, and 90 μL of serum-free medium was added to each tube. Take 10 μL of the viral stock solution to be assayed and add it to the first tube, mix well and take 10 μL and add it to the second tube. Continue the same operation until the last tube. Discard 90 μL of medium in the cell plate, add 90 μL of diluted virus solution, and incubate at 37°C with 5% CO2. 24 h later, add 100 μL of complete medium, and detect the viral titer by RT-PCR after 4 days.
Cells were grown in 24-well plates one day before transfection. Normal culture was performed with 0.5 mL of complete medium without double antibody. Transfection was carried out when the cell density was about 70%; plasmid transfection reagents were configured: 200 ng of shRNA-Wnt4#1, shRNA-Wnt4#2, shRNA-Wnt4#3, and shRNA-NC plasmids (diluted with 1 μL of P3000TM reagent) were incorporated into 150 μL of OPTI-MEM medium, respectively. lipo 3000 0.75 μL was incorporated into 150 μL OPTI-MEM medium, mixed well, and left at room temperature for 5 min; the two were then mixed well and the ultra-clean bench was left to stand for 15 min. During the mixture's resting period, the cell culture solution was pipetted out of the 24-well plate, and rinsed three times with 1×PBS 1 mL. Add 400 μL of OPTI-MEM medium to each well, and put it back to 37°C, 5% CO2 incubator to continue incubation. 15 min later, the medium in the cell culture wells was aspirated out, and the plasmid and transfection reagent mixture was gently added to the 24-well plate according to the

experimental grouping. After shaking and mixing well, the 24-well plate was placed at 37°C for incubation, and the medium was changed to serum-free medium after 6 h. After the cells were stably amplified, the transfection efficiency was verified using PCR assay, and the results were as follows:

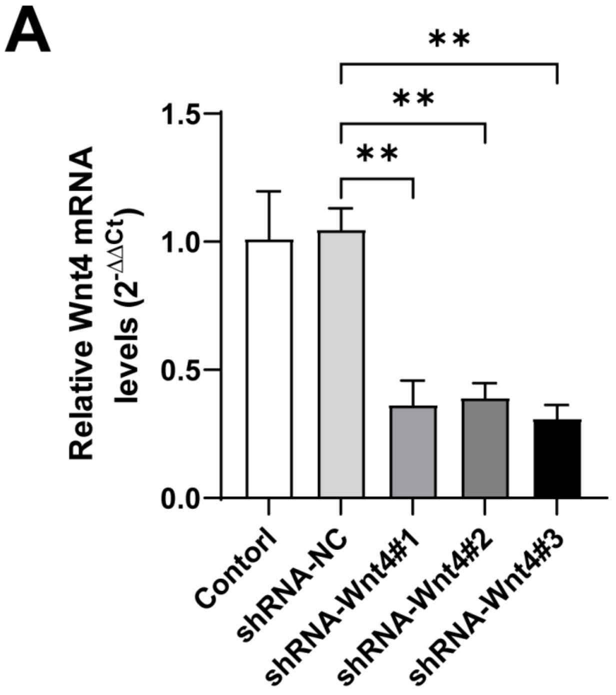

24 h before transfection, 0.5×105 HBVSMC cells were inoculated into each well of a 24-well plate and incubated overnight at 37°C in an incubator with a CO2 concentration of 5%. Serum-free culture medium and Polybrene were mixed to a final concentration of 5 μg/mL. the medium in the 24-well plate was replaced with 0.5 mL of Polybrene-medium mixture per well. Based on the MOI value, the appropriate volume of lentivirus was added to each well. After overnight incubation, the medium was removed and 1 mL of complete medium was added to each well, and incubation was continued at 37°C in an incubator with a CO2 concentration of 5%; after the cells were full-grown, the cells were passaged at a ratio of 1:3. After 48 hours of incubation, the cell culture medium in the petri dish was replaced with Puromycin screening medium containing 2 μg/mL Puromycin, and the incubation was continued. After the cells were able to expand stably in the Puromycin screening medium, the transfection efficiency was

verified using PCR assay, and the results were as follows:

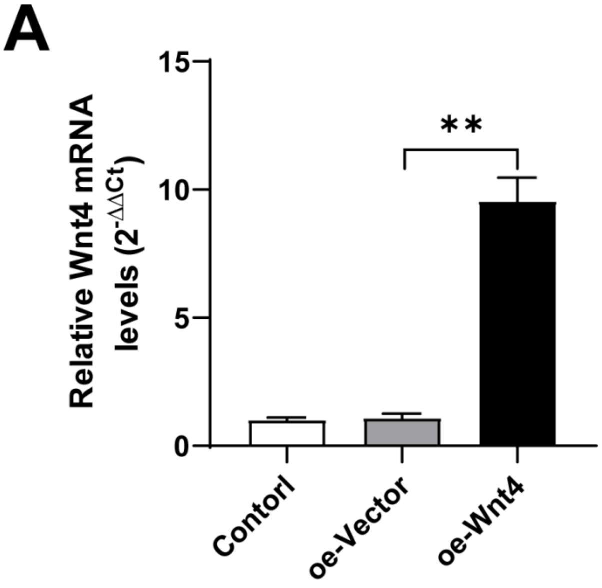

Polymerase chain reaction

Each group was prepared as a cell suspension was inoculated into 6-well plates respectively, the number of cells per well was 5×105. When the cells were fused to 90%, the corresponding treatment was carried out according to the experimental grouping. At the end of the treatment, trypsin was used to collect the cells, centrifuged, and the supernatant was discarded. Add 0.5 mL of Trizol to each well, blow repeatedly until clarified, and transfer to EP tubes, let stand for 5 min. add 0.1 mL of chloroform (1/5 of the volume of Trizol) to each tube, shake vigorously, let stand for 10 min, and then centrifuge at 12,000 rpm at 4°C for 15 min. take the supernatant (do not touch the middle layer), add an equal amount of isopropanol of about 200 μL. shake vigorously, and then stand for 30 min at -20°C to precipitate RNA. Leave for 30 min, precipitate RNA, centrifuge at 12000 rpm 4°C for 10 min, and discard the supernatant. Add 1 mL of 75% ethanol (DEPC water), centrifuge at 7500 rpm 4°C for 5 min, discard the supernatant, and dry naturally for 5-10 min. 10 μL of DEPC water was used to dissolve the RNA, and then the total RNA solution was obtained by blowing and mixing, and 2

μL of the sample was taken, and the mRNA concentration and purity were determined by using Nanodrop 2000.

Take 2 μL of total RNA as a template and configure the reverse transcription reaction system according to the procedure on the kit manual, with a volume of 20 μL of the total system.

The transcription system for reverse transcription is as follows:

Reagents	Volumetric
5×iScript reaction mix	4 μL
iScript reverse transcriptase	1 μL
RNA	2 μL
Nuclease-free water	13 μL
Total	20 μL

The reaction system was placed in a PCR instrument, and the set program was selected to react at 25°C for 5 min, 46°C for 20 min, and 95°C for 1 min. cDNA was cDNA after completion, and stored at -70°C.

The reaction system was configured on the ice box according to the procedure in the reagent instructions, and the system requirements were as follows:

Reagents	Volumetric
TB GreenPremix Ex Taq (Tli RNaseH Plus) (2X)	10 μL
Forward primer	0.4 μL
Reverse primer	0.4 μL
ROX Reference Dye (50X)	0.4 μL
cDNA	2 μL
Ribonuclease free water (Nuclease-free water)	6.8 μL

Total	20 μL

The premixed reagents were added into 8-link tubes, sealed and put into the instrument, and the reaction parameters were set for amplification. The data were processed by the 2-ΔΔCt formula, and the expression level of the target mRNA was obtained using the internal reference gene GAPDH as a reference. The primers used are shown in the table below.

Table. Primers used in Quantitative Real-Time PCR

Primers		Sequence (5'→3')
Wnt4	Forward	CATGAGTCCCCGCTCGTG
	Reverse	TCCATGACTTCCAGGTTCCG
GAPDH	Forward	CCACATCGCTCAGACACCAT
	Reverse	AGTTAAAAGCAGCCCTGGTGA

Western Blot

According to the experimental group, 1 mL of RIPA lysate (KGP702-100, KeyGEN Bio TECH) with PMSF (97064-898, Amresco) was added to each 100 μL compressed volume of cell samples. After sufficient lysis, the lysate was centrifuged at 12000 g for 5 min at 4°C. The supernatant was immediately aspirated into a pre-cooled Eppendorf tube, which was the extracted cellular proteins, and frozen at -80°C for spare use. Protein quantification was performed by BCA method. Finally, 5× loading buffer (WB2001, NCM Biotech) was added to the boiling water bath for 10 min, the sample preparation was completed, and could be stored at -20°C. According to the molecular weight of the proteins to be tested, 10% separating gel and 5% concentrating gel were prepared, and SDS-PAGE gels were filled. After adding 5 mL of prepared separation gel to each gel plate, add isopropyl alcohol to press the gel.

After the gel line was formed (gel completion), the gel rack was placed horizontally and the isopropanol was removed with filter paper. After the concentrated gel is prepared and mixed, at the upper end of the separation gel, inject 2 mL of prepared concentrated gel. immediately insert the comb vertically, and keep the comb horizontal when inserting the comb. After the concentrated gel solidifies, remove the comb and place it in the electrophoresis tank with electrophoresis solution. After adding an appropriate amount of pre-cooled 1× electrophoresis buffer, start to upload the sample (add 5 μL of Marker and the sample in order from left to right in the comb hole). Adjust the sample volume to 60 μg, and electrophoresis was carried out at a constant voltage of 80 V for about 30 min. After the sample entered the separation gel, the voltage was adjusted to 120 V to continue electrophoresis. The electrophoresis was terminated when the target band reached the appropriate position. PVDF membrane (0.2 μm, ISEQ00010, Millipore) was cut to the size of the gel and activated in methanol (10014118, SCRC) for 1 min, and then soaked in transfer buffer, and the filter paper was also soaked in transfer buffer for 15 min, according to the principle of PVDF membrane ≥ gel ≥ filter paper. Make the membrane transfer "sandwich" according to the principle of PVDF membrane ≥ gel ≥ filter paper, make sure that the air bubbles are removed, and then start to transfer the membrane at constant pressure. After completion of the transfer, the membrane was stained with Reichun Red (97063-650, Amresco) s staining solution, followed by TBST (G0001, Servicebio) for 2 washes. The proteins on the membrane were observed. The table below shows the information of the antibodies used.

Table Antibodies and dilution

Antibodies	Dilution (application)	ID	Source
Wnt4	1:500 (WB)	ab262696	Abcam
GSK3β	1:5000 (WB)	ab32391	Abcam
p-GSK3β	1:500 (WB)	AF2016	Affinity Biosciences

β-catenin	1:500 (WB)	BF8016	Affinity Biosciences
active β-catenin	1:1000 (WB)	ab305261	Abcam
Tau	1:500 (WB)	AF6141	Affinity Biosciences
p-Tau	1:500 (WB)	PA5-117230	Thermo Fisher Scientific
PSEN1	1:500 (WB)	AF0245	Affinity Biosciences
TUBA	1:500 (WB)	DF14454	Affinity Biosciences
TUBB	1:1000 (WB)	BF0716	Affinity Biosciences
Acetyl-α-tubulin	1:2000 (WB)	ab179484	Abcam
α-tubulin	1:1000 (WB)	ab52866	Abcam
eNOS	1:1000 (WB)	ab252439	Abcam
p-eNOS	1:1000 (WB)	ab215717	Abcam
GAPDH	1:1000 (WB)	ab8245	Abcam

Immunofluorescence staining

Soak coverslips (22×22 mm) with 75% alcohol for 2 h, and then place them in 6-well plates after waving away the alcohol; inoculate the cells with 5×105 cells/well in 6-well plates according to the experimental grouping, and then place them in culture at 37°C under 5% CO2; observe them under an inverted microscope (Olympus), and then discard the supernatant when the cell proliferation reaches 80%; add 500 μL 4% paraformaldehyde for 15 min; wash with PBS solution 3 times, each time for 5 min; add 0.1% TritonX-100, incubate for 10 min at room temperature, and wash with PBS solution 3 times, each time for 5 min. paraformaldehyde for 15 min; wash with PBS 3 times, 5 min each time; add 0.1% TritonX-100, incubate for 10 min at room

temperature, wash with PBS 3 times, 5 min each time; seal the cells with serum sealing solution for 30 min at room temperature; discard the sealing solution, and add Anti-β-Tubulin antibody (ab18207, Abcam) (final concentration 1 μg/mL) and incubate at 4°C overnight; at the end of the incubation, remove and wash with PBS 3 times, 5 min each time; add Goat Anti-Rabbit IgG H&L (ab6721, Abcam) (Alexa Fluor® 488) (dilution ratio 1:1000) and incubate for 1 h, avoiding light. The cells were washed three times with PBS solution for 5 min each time; the cells were blocked with serum blocking solution for 30 min at room temperature; the blocking solution was discarded, and Anti-F-actin antibody (ab205, Abcam) (final concentration of 1 μg/mL) was added, and incubated overnight at 4°C; at the end of the incubation, the cells were removed and washed three times with PBS solution for 5 min each time; the cells were incubated for 1 h at room temperature; the cells were incubated for 1 h at room temperature, and the cells were incubated for 5 min at room temperature. Anti-Mouse IgG H&L (ab6728, Abcam) (Alexa Fluor® 594) (dilution ratio of 1:1000) was incubated at 4°C overnight, and washed with PBS three times for 5 min each time; the film was sealed with a drop of DAPI, and the excess liquid was drained off; the film was observed under a laser confocal microscope (Zeiss LSM), and the field of view was randomly selected for photographs at a magnification of 200 ×. The image was photographed under a laser confocal microscope (Zeiss LSM) at 200× magnification.

Flow Cytometry

Flow cytometry was used to detect apoptosisusing Annexin V-FITC/PI Apoptosis Detection Kit (KGA108-1, KeyGEN) Cells were sequentially grouped according to the corresponding treatment and then lubricated twice with PBS; cells were treated with EDTA-free trypsin (15090046, Gibco, Grand Island). Cells were treated with EDTA-free trypsin (15090046, Gibco, Grand Island), and the digestion was terminated with complete culture solution when the band cells were deformed and the intercellular junctions disappeared; the cell suspension was transferred to a sterile centrifuge tube, centrifuged at 800 rpm for 5 min, and the supernatant was discarded; the cells were resuspended by adding 0.5 mL of staining buffer; 5 μL of FITC staining

solution and 5 μL of PI staining solution were added, and the mixture was mixed well by gentle blowing, and the cells were incubated for 15 min under room conditions with the protection of light; the cells were incubated for 15 min under room conditions with flow cytometry (Beckman DxFlex) for detection.

ROS assay

Experiments were performed using the ROS detection kit (S0033S, Beyotime), and DCFH-DA was diluted with serum-free culture medium according to 1:1000, with a final concentration of 10 μM. Cells were collected and suspended in diluted DCFH-DA at a concentration of 1 × 106 cells/ml, and incubated in a cell culture incubator at 37°C for 20 min protected from light. The cells were washed three times with serum-free cell culture medium to fully remove the DCFH-DA that did not enter the cells.Observation was performed under a laser confocal microscope (Zeiss LSM), and the field of view was randomly selected for photographing at 200× filming magnification.

Cell Scratching Assay

Horizontal lines were evenly scribed on the back of 6-well plates with a UV-neutralized marker pen, approximately one line every 0.5-1 cm across the wells, with at least 5 lines across each well. Cells in the logarithmic growth phase were digested into single-cell suspensions with trypsin (15090046, Gibco, Grand Island), and the concentration of the cell suspension was adjusted by adding the appropriate medium, and 2 mL of cells at a density of 6 × 105 cells/mL were seeded in the scribed 6-well plate to ensure that the cells could grow to full size the next day, for a final total of 2 mL of medium per well. in accordance with the Grouping Requirements 37 °C, 5% CO2 cell incubator culture. 24 h later when the cells were observed under the microscope to be adherent to the wall and uniformly distributed, the cells were scratched with a sterilized 200 μL tip perpendicular to the marked horizontal line behind them in the ultra-cleaning table (SW-CJ-2FD, Suzhou Purification Equipment Co., Ltd.), the tip of the tip of the tip of the gun should be perpendicular to the line, and it can not be tilted. The cells were washed with PBS for 3 times, the scratched cells were removed, serum-free medium was added, and the cells were incubated in

an incubator at 37°C, 5% CO_2. Photographs were taken at a magnification of 100× to ensure that the scratches were centered and perpendicular, with attention paid to the consistent background, and the samples were sampled in accordance with the time points, and photographs were taken for recording.

Statistical analysis

Data were analyzed and graphed using Graphpad Prism 9 (Version 9.4.0) and collated using Adobe Illustrator 2022 (Version 2022). All data were expressed as means ± SD, and statistical differences between groups were analyzed using one-way ANOVA and Tukey's test, with P values less than 0.05 considered significant.

Results

Participants and samples

This study included 100 patients who were diagnosed with MMD between January 2020 and August 2022. Peripheral blood samples were collected from all 100 patients. In the discovery group, DIA quantification proteomics were performed on the peripheral blood of 40 patients with MMD (20 HEM and 20 IS) and 20 HC. DIA results are provided in the Supplementary material (Table S2). To study the effect of proteins in the peripheral serum of patients with MMD (20 HEM and 20 IS) on MMD hemorrhage, a DIA proteomic approach was used to identify the differences in protein expression between peripheral sera of HEM, IS, and HC. The results showed significantly higher levels of Wnt4 expression in the serum of the HEM than those in the IS and HC.

Upregulated Wnt4 protein expression from serum from patients with hemorrhagic MMD

In order to search for differential genes between HEM and IS, in the discovery group, the gene sets of 40 patients with MMD (20 HEM, 20 IS) were analyzed for differences. Principal component analysis shows that based on gene expression levels, the HEM group was distinguished from the IS group (Figure2 A). Volcano plots showed that the Wnt4 was one of the most significantly different and statistically

significant genes in the IS group compared to the HEM group (Figure2 B). In order to explore the role of the differential genes in MMD, the differential genes were functionally analyzed. The chordal graph shows that Wnt4 is significantly associated with positive regulation of cell substrate adhesion, immature t cell proliferation in thymus, immature t cell proliferation and positive regulation of cell matrix adhesion (Figure2 C). To sum up, the expression levels of Wnt4 differed significantly between the HEM and IS groups. In addition to that, it was strongly correlated due to multiple important functions. Therefore, Wnt4 was selected for follow-up studies and to explore its specific role in hemorrhagic Moyamoya disease. In the validation group, Wnt4 expression levels were measured by ELISA in peripheral blood of 60 patients with MMD (30 HEM, 30 IS) and 20 HC. The results indicated a slight increase in Wnt4 expression levels in the serum of patients in the IS group compared to the HC group, and a more significant increase in Wnt4 expression levels in the serum of patients in the HEM group. Furthermore, there was a significant increase in Wnt4 expression levels in the serum of patients in the HEM group compared to those in the IS group (Figure2 D).

Upregulated Wnt4 has multiple negative effects on HBVSMC, including inhibition of cellular microtubules and the cytoskeleton

To explore the effect of upregulated Wnt4 on vascular smooth muscle cells (VSMC) in patients with hemorrhagic MMD, a lentiviral Wnt4 overexpression cell model was constructed using HBVSMC. Subsequently, morphological alterations of the cells were observed using light microscopy, and immunofluorescence staining was conducted with Anti-β-Tubulin and Anti-F-actin antibodies. The results showed altered cell morphology, decreased microtubule density, and decreased cytoskeletal stability in HBVSMC in the oe-Wnt4 group compared to the oe-Vector group (Figure3 A). ROS levels in HBVSMC were detected with a ROS detection kit (S0033S), and the results showed that the ROS content increased significantly in HBVSMC in the oe-Wnt4 group compared to the oe-Vector group (Figure S1A). In terms of the cellular level, upregulated Wnt4 had a negative effect on the microtubules and cytoskeleton of HBVSMC. The levels of eNOS and p-eNOS were

detected by western blot assay, and the results showed that the phosphorylation level of eNOS was significantly reduced in the oe-Wnt4 group compared to that in the oe-vector group (Figure S1B). The effect of overexpression of Wnt4 on proliferation and apoptosis of HBVSMC was detected using flow cytometry and scratch assay, which showed that apoptosis of HBVSMC in the oe-Wnt4 group increased significantly, and cell proliferation reduced significantly compared to the oe-Vector group (Figure S1C, S1D). To continue to investigate the specific mechanism of these effects, the expression levels of Wnt4, p-GSK3β, tau, β-catenin, p-tau, PSEN1, TUBA, and TUBB in HBVSMC were examined using a western blot assay. The western blot assay results showed that compared to the oe-Vector group, there were no significant changes in the expression level of tau in HBVSMC cells in the oe-Wnt4 group, the expression levels of Wnt4, β-catenin, active-β-catenin, PSEN1, TUBA, TUBB, and the phosphorylation level of tau increased significantly, and the phosphorylation level of GSK3β decreased significantly (Figure3 B). To explore the specific effects of changes in proteins, such as Wnt4, p-GSK3β, tau, β-catenin, p-tau, PSEN1, TUBA, and TUBB on cellular microtubules, total microtubule levels and acetylated microtubule levels in HBVSMC were examined using a western blot assay. The western blot assay results showed that compared to the oe-Vector group, acetyl-α-tubulin and α-tubulin levels were reduced in HBVSMC cells of the oe-Wnt4 group, α-tubulin acetylation was reduced, and microtubule stability was decreased (Figure3 C). In summary, we hypothesized that upregulation of the Wnt4 protein causes cellular injury by affecting the stability of cellular microtubules and the total microtubule level.

The negative effects of serum from hemorrhagic patients with MMD on HBVSMC cultivation showed improvement after Wnt4 knockdown

To explore the morphological and phenotypic changes of HBVSMC in the HEM group after knockdown of Wnt4, multiple groups of in vitro experiments were carried out between the shRNA-NC+HEM group and the shRNA-Wnt4+HEM group. Fluorescence immunostaining using anti-β-Tubulin antibody with anti-F-actin antibody showed increased microtubule density, improved cytoskeletal stability, and cell

morphology close to normal cells in HBVSMC cells of the shRNA-Wnt4+HEM group compared to the shRNA-NC+HEM group (Figure3 D). The ROS detection kit (S0033S) was used to detect the ROS level of HBVSMC in the HEM group after the knockdown of Wnt4; the results showed that ROS levels decreased significantly in HBVSMC in the shRNA-Wnt4+HEM group compared to the shRNA-NC+HEM group (Figure3 D). Scratch assay and flow cytometry were used to detect cell proliferation ability and apoptosis levels of HBVSMC in the HEM group after Wnt4 knockdown. The results showed that the cell proliferation ability of HBVSMC in the shRNA-Wnt4+HEM group increased significantly and the apoptosis level decreased significantly compared to that of the shRNA-NC+HEM group (Figure3 E, F). To explore the specific changes of downstream molecules in HBVSMC of the HEM group after knockdown of Wnt4, the expression levels of acetyl-α-tubulin, α-tubulin, and p-eNOS were detected using western blot assay, and the results showed that compared to shRNA-NC+HEM group, acetyl-α tubulin and α-tubulin levels increased significantly and p-eNOS levels were significantly elevated (Figure3 G).

Wnt4 inhibitor DKK-1 reverses the negative effects of overexpression of Wnt4-induced decrease in cytoskeletal density and microtubule stability by blocking GSK3β/β-catenin signaling pathway

To investigate the specific effects of the Wnt4 inhibitor DKK-1 on each signaling molecule of the GSK3β/β-catenin signaling pathway, a western blot assay was used to detect the expression levels of Wnt4, GSK3β, p-GSK3β, β-catenin, active β-catenin, p-tau, PSEN1, TUBA, tau and TUBB. The results showed that, compared with the oe-Vector group, there was no significant change in the expression levels of GSK3β and tau in HBVSMC cells in the oe-Wnt4 group, and the expression levels of Wnt4, β-catenin, active-β-catenin, PSEN1, TUBA, TUBB and the phosphorylation level of tau increased significantly, and the phosphorylation level of GSK3β was level was significantly reduced. Compared to the oe-Wnt4 group, there was no significant change in the expression levels of GSK3β and tau in HBVSMC cells in the oe-Wnt4+DKK-1 group, and the expression levels of Wnt4, β-catenin, active-β-catenin, PSEN1, TUBA, TUBB, and the phosphorylation level of tau were decreased to different degrees, and

the phosphorylation level of GSK3β increased significantly. β phosphorylation level increased significantly (Figure S2). Fluorescence immunostaining using anti-β-Tubulin antibody with anti-F-actin antibody showed a decrease in cellular microtubule density, a decrease in cytoskeletal stability and a change in cellular morphology in HBVSMC cells in the oe-Wnt4 group compared to the oe-Vector group. Compared to the oe-Wnt4 group, the density of cellular microtubules in HBVSMC in the oe-Wnt4+DKK-1 group increased, cytoskeletal stability was improved, and cellular morphology tended to be normalized (Figure4 A). The ROS levels were detected using the ROS detection kit (S0033S), which shows a significant decrease in ROS content in the oe-Wnt4+DKK-1 group compared to the oe-Wnt4 group (Figure4 A). The expression levels of acetyl-α-tubulin, α-tubulin, eNOS and p-eNOS were detected using western blot assay, and the results showed that compared to the oe-Vector group, the levels of acetyl-α-tubulin and α-tubulin in HBVSMC cells in the oe-Wnt4 group were significantly decreased, α-tubulin acetylation decreased, microtubule stability decreased, eNOS content did not change significantly, and p-eNOS content decreased significantly. Compared to the oe-Wnt4 group, acetyl-α-tubulin and α-tubulin contents increased significantly in HBVSMC cells in the oe-Wnt4+DKK-1 group, the degree of α-tubulin acetylation was not significantly changed, eNOS content was not significantly changed, and p-eNOS content decreased significantly (Figure 4 B, C). Cell proliferation ability and apoptosis level were detected using scratch assay and flow cytometry, respectively, showing that apoptosis increased significantly and cell proliferation weakened significantly in HBVSMC in the oe-Wnt4 group compared to the oe-Vector group. Compared to the oe-Wnt4 group, HBVSMC apoptosis was significantly reduced, and cell proliferation enhanced significantly in the oe-Wnt4+DKK-1 group (Figure 4 D, E).

Figure S1: The effects of the knockout of Wnt4 on the ROS, eNOS, cell proliferation and cell migration of HBVSMC cells which were cultured with HEM.

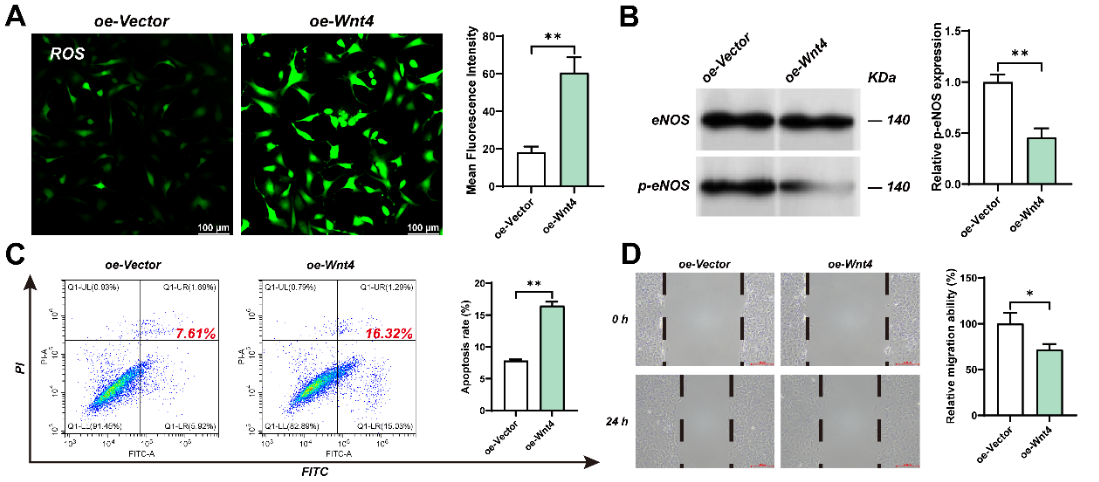

HBVSMC cells were treated with oe-Vector or oe-Wnt4.

A: The ROS levels were detected by DCFH-DA probe fluorescence microscopy. Bar=100 μm. Results were mean ± SD for three individual experiments. *p<0.05, **p<0.01.

B: HBVSMC cells were treated with oe-Vector or oe-Wnt4. The expression of eNOS and from the indicated group was detected by western blot assay. Results were mean ± SD for three individual experiments. *p<0.05, **p<0.01.

C: Flow Cytometric Analysis of cell apoptosis in HBVSMC cells. Results were mean ± SD for three individual experiments. *p<0.05, **p<0.01.

D: Cell migration was detected by scratch assay. Bar=200 μm. Results were mean ± SD for three individual experiments. *p<0.05, **p<0.01.

Figure S2: The effect of Wnt4 overexpression on proteins of HBVSMC cells after addition of inhibitor DKK-1.

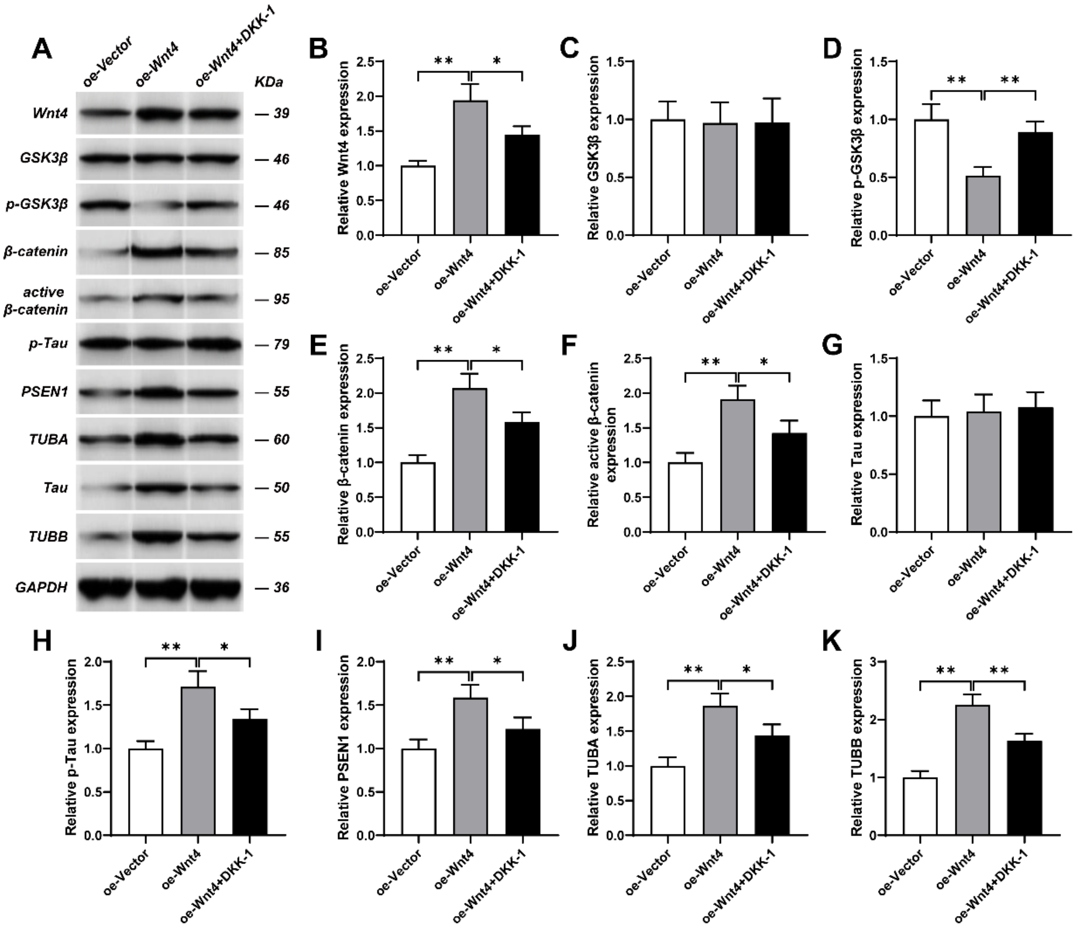

HBVSMC cells were treated with oe-Vector or oe-Wnt4, then one group of cells transfected with oe-Wnt4 was cultured in SMCM medium with 100 μg/L DKK-1.

A~K: The expression of Wnt4, GSK3β, p-GSK3β, β-catenin, active-β-catenin, p-Tau, PSEN1, TUBA, Tau and TUBB from the indicated group was detected by western blot assay. The ordinate is the protein name. The abscissa is the group name. Results were mean ± SD for three individual experiments. *p<0.05, **p<0.01.

Table S1: Clinical characteristics and sample information of the patients with MMD.

No. of Patients	Groups	Sex	Age (years)	Hypertension	Diabetes	Coronary heart disease	Hyperlipidemia	Smoking history	Alcohol taking	Duration of symptoms (months)	Suzuki stage	Family history	Usage (Sample)
P1	HEM	M	44	YES	NO	NO	NO	NO	NO	4	L3/R1	NO	DIA (Serum)
P2	HEM	M	52	NO	NO	NO	NO	NO	NO	14	L3/R0	NO	DIA (Serum)
P3	HEM	F	35	NO	NO	NO	NO	NO	NO	6	L5/R4	NO	DIA (Serum)
P4	HEM	F	43	NO	NO	NO	NO	NO	NO	5	L5/R1	NO	DIA (Serum)
P5	HEM	M	17	NO	NO	NO	NO	NO	NO	42	L4/R5	NO	DIA (Serum)
P6	HEM	F	34	YES	NO	NO	NO	NO	NO	8	L4/R3	NO	DIA (Serum)
P7	HEM	M	27	NO	NO	NO	NO	NO	NO	5	L2/R3	NO	DIA (Serum)
P8	HEM	F	19	NO	NO	NO	NO	NO	NO	4	L1/R1	NO	DIA (Serum)
P9	HEM	F	43	NO	NO	NO	NO	NO	NO	2	L2/R3	NO	DIA (Serum)
P10	HEM	M	32	NO	NO	NO	NO	NO	NO	5	L3/R3	NO	DIA (Serum)
P11	HEM	F	26	NO	NO	NO	NO	NO	NO	4	L2/R1	NO	DIA (Serum)
P12	HEM	F	38	NO	NO	NO	NO	NO	NO	11	L3/R6	NO	DIA (Serum)
P13	HEM	M	23	NO	NO	NO	NO	NO	NO	1.5	L1/R2	NO	DIA (Serum)
P14	HEM	F	16	YES	NO	NO	NO	NO	NO	12	L2/R4	NO	DIA (Serum)
P15	HEM	F	42	NO	NO	NO	NO	NO	NO	5	L3/R3	NO	DIA (Serum)
P16	HEM	M	32	NO	NO	NO	NO	NO	NO	6	L0/R2	NO	DIA (Serum)
P17	HEM	M	40	NO	NO	NO	NO	NO	NO	24	L2/R2	NO	DIA (Serum)
P18	HEM	M	28	YES	NO	NO	NO	YES	NO	4	L3/R2	NO	DIA (Serum)
P19	HEM	M	40	NO	NO	NO	NO	YES	YES	2	L3/R3	NO	DIA (Serum)
P20	HEM	F	46	NO	NO	NO	NO	NO	NO	120	L3/R2	NO	DIA (Serum)
P21	IS	F	42	NO	NO	NO	NO	NO	NO	72	L5/R3	NO	DIA (Serum)
P22	IS	F	32	YES	NO	NO	NO	NO	NO	18	L2/R2	NO	DIA (Serum)
P23	IS	F	39	NO	NO	NO	NO	NO	NO	72	L4/R3	NO	DIA (Serum)
P24	IS	M	31	NO	NO	NO	NO	NO	NO	12	L3/R3	NO	DIA (Serum)
P25	IS	F	31	NO	NO	NO	NO	NO	NO	3	L2/R3	NO	DIA (Serum)
P26	IS	F	34	NO	NO	NO	NO	NO	NO	4	L2/R3	NO	DIA (Serum)
P27	IS	F	38	NO	NO	NO	NO	NO	NO	48	L2/R2	NO	DIA (Serum)
P28	IS	M	34	NO	NO	NO	NO	2	NO	1	L2/R3	NO	DIA (Serum)

P29	IS	M	15	NO	NO	NO	NO	NO	NO	144	L3/R5	NO	DIA (Serum)
P30	IS	F	19	NO	NO	NO	NO	NO	NO	1	L2/R3	NO	DIA (Serum)
P31	IS	M	38	NO	NO	NO	NO	NO	NO	21	L5/R2	NO	DIA (Serum)
P32	IS	M	38	NO	NO	NO	NO	NO	NO	3.5	L5/R3	NO	DIA (Serum)
P33	IS	M	31	NO	NO	NO	NO	NO	YES	24	L0/R2	NO	DIA (Serum)
P34	IS	F	30	NO	NO	NO	NO	NO	NO	24	L1/R3	NO	DIA (Serum)
P35	IS	M	38	NO	NO	NO	NO	YES	NO	2	L2/R3	NO	DIA (Serum)
P36	IS	M	20	NO	NO	NO	NO	NO	NO	3	L2/R4	NO	DIA (Serum)
P37	IS	F	34	YES	NO	NO	NO	NO	NO	2	L3/R3	NO	DIA (Serum)
P38	IS	F	43	NO	NO	NO	NO	NO	NO	3	L3/R2	NO	DIA (Serum)
P39	IS	M	35	NO	NO	NO	NO	NO	NO	5	L4/R2	NO	DIA (Serum)
P40	IS	F	35	NO	NO	NO	NO	NO	NO	12	L1/R4	NO	DIA (Serum)
P41	HEM	F	56	NO	NO	NO	YES	NO	NO	120	L3/R3	NO	ELISA (Serum)
P42	HEM	F	52	NO	NO	NO	NO	NO	NO	72	L4/R3	NO	ELISA (Serum)
P43	HEM	F	16	NO	NO	NO	NO	NO	NO	12	L4/R2	NO	ELISA (Serum)
P44	HEM	M	50	NO	NO	NO	NO	NO	NO	6	L3/R2	NO	ELISA (Serum)
P45	HEM	F	16	NO	NO	NO	NO	NO	NO	4	L3/R3	NO	ELISA (Serum)
P46	HEM	M	49	NO	NO	NO	NO	NO	NO	12	L4/R3	NO	ELISA (Serum)
P47	HEM	M	35	NO	NO	NO	NO	NO	NO	6	L3/R3	NO	ELISA (Serum)
P48	HEM	F	47	NO	NO	NO	NO	NO	NO	7	L4/R3	NO	ELISA (Serum)
P49	HEM	M	18	NO	NO	NO	NO	NO	NO	5	L2/R2	NO	ELISA (Serum)
P50	HEM	M	16	NO	NO	NO	NO	NO	NO	1	L2/R1	NO	ELISA (Serum)
P51	HEM	M	19	NO	NO	NO	NO	NO	NO	4	L3/R3	NO	ELISA (Serum)
P52	HEM	M	41	NO	NO	NO	NO	NO	NO	4	L3/R3	NO	ELISA (Serum)
P53	HEM	M	42	NO	NO	NO	NO	NO	NO	24	L3/R3	NO	ELISA (Serum)
P54	HEM	F	57	NO	NO	NO	NO	NO	NO	3	L3/R3	NO	ELISA (Serum)
P55	HEM	M	38	NO	NO	NO	NO	NO	NO	4	L4/R3	NO	ELISA (Serum)
P56	HEM	F	32	NO	NO	NO	NO	NO	NO	10	L3/R3	NO	ELISA (Serum)
P57	HEM	F	34	NO	NO	NO	NO	NO	NO	6	L3/R3	NO	ELISA (Serum)
P58	HEM	F	40	NO	NO	NO	NO	NO	NO	18	L4/R4	NO	ELISA (Serum)
P59	HEM	M	30	NO	NO	NO	NO	NO	NO	12	L3/R3	NO	ELISA (Serum)
P60	HEM	F	19	NO	NO	NO	NO	NO	NO	12	L3/R3	NO	ELISA (Serum)
P61	HEM	M	23	NO	NO	NO	NO	NO	NO	1.5	L2/R2	NO	ELISA (Serum)

P62	HEM	M	27	NO	NO	NO	NO	NO	NO	12	L3/R2	NO	ELISA (Serum)
P63	HEM	M	28	NO	NO	NO	NO	NO	NO	4	L3/R4	NO	ELISA (Serum)
P64	HEM	M	32	NO	NO	NO	NO	NO	NO	5	L4/R4	NO	ELISA (Serum)
P65	HEM	M	44	NO	NO	NO	NO	NO	NO	12	L3/R3	NO	ELISA (Serum)
P66	HEM	M	52	NO	NO	NO	NO	YES	NO	12	L3/R3	NO	ELISA (Serum)
P67	HEM	M	40	NO	NO	NO	NO	NO	NO	4	L0/R3	NO	ELISA (Serum)
P68	HEM	F	16	YES	NO	NO	NO	NO	NO	12	L2/R4	NO	ELISA (Serum)
P69	HEM	F	26	NO	NO	NO	NO	NO	NO	4	L3/R1	NO	ELISA (Serum)
P70	HEM	F	34	YES	NO	NO	NO	NO	NO	8	L4/R3	NO	ELISA (Serum)
P71	IS	M	55	NO	NO	NO	NO	NO	NO	8	L3/R3	NO	ELISA (Serum)
P72	IS	M	35	NO	NO	NO	NO	NO	NO	12	L3/R3	NO	ELISA (Serum)
P73	IS	F	34	NO	NO	NO	NO	NO	NO	36	L3/R3	NO	ELISA (Serum)
P74	IS	F	19	NO	NO	NO	NO	NO	NO	24	L3/R2	NO	ELISA (Serum)
P75	IS	F	35	NO	NO	NO	NO	NO	NO	60	L3/R3	NO	ELISA (Serum)
P76	IS	M	11	NO	NO	NO	NO	NO	NO	24	L2/R3	NO	ELISA (Serum)
P77	IS	F	40	NO	NO	NO	NO	NO	NO	24	L3/R3	NO	ELISA (Serum)
P78	IS	F	25	NO	NO	NO	NO	NO	NO	120	L2/R3	NO	ELISA (Serum)
P79	IS	M	19	NO	NO	NO	NO	NO	NO	24	L0/R3	NO	ELISA (Serum)
P80	IS	M	7	NO	NO	NO	NO	NO	NO	24	L3/R3	NO	ELISA (Serum)
P81	IS	F	45	NO	NO	NO	NO	NO	NO	24	L3/R3	NO	ELISA (Serum)
P82	IS	M	41	NO	NO	NO	NO	NO	NO	12	L4/R4	NO	ELISA (Serum)
P83	IS	F	57	NO	NO	NO	NO	NO	NO	6	L3/R3	NO	ELISA (Serum)
P84	IS	M	25	NO	NO	NO	NO	NO	NO	15	L3/R3	NO	ELISA (Serum)
P85	IS	F	53	NO	NO	NO	NO	NO	NO	8	L3/R4	NO	ELISA (Serum)
P86	IS	F	38	NO	NO	NO	NO	NO	NO	8	L4/R3	NO	ELISA (Serum)
P87	IS	F	55	NO	NO	NO	NO	NO	NO	10	L3/R3	NO	ELISA (Serum)
P88	IS	M	37	3	NO	NO	NO	NO	NO	10	L3/R4	NO	ELISA (Serum)
P89	IS	M	41	NO	NO	NO	NO	NO	NO	5	L3/R4	NO	ELISA (Serum)
P90	IS	M	8	NO	NO	NO	NO	NO	NO	12	L4/R3	NO	ELISA (Serum)
P91	IS	F	51	NO	NO	NO	NO	NO	NO	4	L3/R3	NO	ELISA (Serum)
P92	IS	M	4	NO	NO	NO	NO	NO	NO	24	L3/R3	NO	ELISA (Serum)
P93	IS	F	30	NO	NO	NO	NO	NO	NO	1	L4/R3	NO	ELISA (Serum)
P94	IS	F	24	NO	NO	NO	NO	NO	NO	3	L3/R3	NO	ELISA (Serum)

P95	IS	M	38	NO	NO	NO	NO	NO	NO	21	L5/R2	NO	ELISA (Serum)
P96	IS	F	19	NO	NO	NO	NO	NO	NO	1	L2/R3	NO	ELISA (Serum)
P97	IS	F	34	NO	NO	NO	NO	NO	NO	4	L2/R3	NO	ELISA (Serum)
P98	IS	F	43	NO	NO	NO	NO	NO	NO	3	L3/R2	NO	ELISA (Serum)
P99	IS	M	15	NO	NO	NO	NO	NO	NO	144	L3/R5	NO	ELISA (Serum)
P100	IS	M	34	NO	NO	NO	NO	YES	NO	1	L2/R3	NO	ELISA (Serum)

MMD, moyamoya disease. HEM, hemorrhagic moyamoya disease; IS, ischemic moyamoya disease. M, male; F, female. L, left; R, right. DIA, data-independent acquisition mass spectrometry; ELISA, enzyme-linked immunosorbent assays. Duration of symptoms indicates the duration from the first symptom until the hospitalization.

Table S2: List of differentially expressed proteins in DIA data (The top ten most obvious differences were sorted according to Log2FC (IS / HEM).).

**Protein ID**	**Protein name**	**Gene Symbol**	**Gene ID**	**Log2FC (IS / HEM)**	**Log2FC (HEM/ HC)**	**Log2FC (IS / HC)**	**Qvalue (IS / HEM)**	**Qvalue (HEM / HC)**	**Qvalue (IS / HC)**
B4E3S6	B4E3S6	C7	730	-65.04835	1.259235	-63.78911	0.1254139	0.9761536	0.1376591
Q13747	Q13747	SERPINA1	5265	-4.442656	NA	NA	NA	NA	NA
A0A2U8J975	A0A2U8J975	FAM30A	9834	-3.124965	0.7876699	-2.337296	0.2258299	0.7591559	0.3640368
P02679	FIBG	FGG	2266	-2.885537	3.581335	0.6957981	0.1829335	0.4026549	0.8698325
Q9H254	SPTN4	SPTBN4	57731	-2.418727	NA	NA	NA	NA	NA
Q14508	WFDC2	WFDC2	10406	-2.391458	NA	NA	0.009709223	NA	NA
P56705	WNT4	WNT4	54361	-2.190517	2.582049	0.391532	0.03045608	0.07248056	0.7556595
Q9NSD6	Q9NSD6	LOC107985543	107985543	-2.148858	1.176563	-0.9722946	0.3384086	0.3892497	0.6637057
P06576	ATPB	ATP5F1B	506	-1.932519	NA	NA	0.001069066	NA	NA
Q15126	PMVK	PMVK	10654	-1.917233	-0.3936866	-2.31092	0.002436313	0.2358995	0.001086127

HEM, hemorrhagic moyamoya disease; IS, ischemic moyamoya disease; HC, health control; DIA, data-independent acquisition mass spectrometry.

1 HASHIMOTO, N. Guidelines for diagnosis and treatment of moyamoya disease (spontaneous occlusion of the circle of Willis）. *Neurol Med Chir (Tokyo)*, doi:10.2176/nmc.52.245 (2012).
